# Luminescence from Droplet-Etched GaAs Quantum Dots at and Close to Room Temperature

**DOI:** 10.3390/nano11030690

**Published:** 2021-03-10

**Authors:** Leonardo Ranasinghe, Christian Heyn, Kristian Deneke, Michael Zocher, Roman Korneev, Wolfgang Hansen

**Affiliations:** Center for Hybrid Nanostructures (CHyN), University of Hamburg, Luruper Chaussee 149, 22761 Hamburg, Germany; heyn@physnet.uni-hamburg.de (C.H.); kdeneke@physnet.uni-hamburg.de (K.D.); mzocher@physnet.uni-hamburg.de (M.Z.); rkorneev@physnet.uni-hamburg.de (R.K.); hansen@physnet.uni-hamburg.de (W.H.)

**Keywords:** semiconductor, nanostructuring, quantum dot, self-assembly, droplet etching, room temperature, photoluminescence

## Abstract

Epitaxially grown quantum dots (QDs) are established as quantum emitters for quantum information technology, but their operation under ambient conditions remains a challenge. Therefore, we study photoluminescence (PL) emission at and close to room temperature from self-assembled strain-free GaAs quantum dots (QDs) in refilled AlGaAs nanoholes on (001)GaAs substrate. Two major obstacles for room temperature operation are observed. The first is a strong radiative background from the GaAs substrate and the second a significant loss of intensity by more than four orders of magnitude between liquid helium and room temperature. We discuss results obtained on three different sample designs and two excitation wavelengths. The PL measurements are performed at room temperature and at *T* = 200 K, which is obtained using an inexpensive thermoelectric cooler. An optimized sample with an AlGaAs barrier layer thicker than the penetration depth of the exciting green laser light (532 nm) demonstrates clear QD peaks already at room temperature. Samples with thin AlGaAs layers show room temperature emission from the QDs when a blue laser (405 nm) with a reduced optical penetration depth is used for excitation. A model and a fit to the experimental behavior identify dissociation of excitons in the barrier below *T* = 100 K and thermal escape of excitons from QDs above *T* = 160 K as the central processes causing PL-intensity loss.

## 1. Introduction

Semiconductor quantum dots (QDs) are central building blocks for advanced applications. These range from QD-based lasers with low threshold currents [1], over quantum information processing and quantum cryptography [2,3], exploiting QDs, for example, as single [4] and entangled photon sources [5] to further optoelectronic applications, such as solar cells [6,7] and optical amplifiers [8]. However, for the commercial use of these devices, operation at room temperature ($T_{R}$) represents an important challenge.

Several advances have been made towards the development of QDs for optical applications that can be integrated, for example, into quantum photonic circuits [9,10]. In particular, epitaxial self-assembled QDs grown by molecular beam epitaxy (MBE) have been established for this purpose. The most popular mechanisms for the spontaneous accumulation of QD material are the strain-induced growth in the Stranski–Krastanov mode [11,12,13,14,15] and droplet epitaxy in the Volmer–Weber mode [16]. However, the former works only with lattice-mismatched material combinations and produces QDs that are substantially strained [17], usually leading to a strong fine-structure splitting [18]. Furthermore, they are affected by unintentional intermixing with substrate material and thus have a poorly controlled QD composition [19,20]. The droplet epitaxy method, on the other hand, produces strain-free QDs [21,22,23], but their fabrication requires an advanced growth temperature program to improve optical quality and flushing techniques for narrow-sized uniformity [24].

In the following, QDs generated by an alternative droplet-based technique are addressed. This technique, local droplet etching (LDE), allows for the creation of unstrained, pure and highly uniform QDs [19]. LDE QDs have been widely studied at helium temperatures and shown to have state-of-the-art optical properties, with exciton-peak line width down to 25 μeV [19,25,26], neutral exciton fine-structure splitting as low as 4.5 μeV [26] and single photon emission proven by a second-order correlation function of 0.01 [27].

While epitaxially grown QDs have interesting optical properties at cryogenic temperatures, not much study is present in the literature about the optical emission under ambient conditions. As a step forward, the present article studies QDs at room temperature ($T_{R}$) and at slightly lower temperatures. Strong emission at $T_{R}$, with clearly resolved QD shell-structure, is demonstrated from an ensemble of GaAs QDs fabricated using LDE. Central issues for QD operation at and close to $T_{R}$ are a crucial reduction in the QD emission intensity, as well as an increasing substrate background. Both effects are modeled and the results are compared with the experimental data.

## 2. Methods

### 2.1. Sample Fabrication

The central method for GaAs QD fabrication is local droplet etching during solid-source MBE [28,29,30,31,32]. LDE is fully compatible with the demanding requirements of the MBE technique and allows a self-assembled patterning of semiconductor surfaces without any lithographic steps. For the present samples on semi insulating (001)GaAs wafers, Al droplets are used to drill nanoholes into AlGaAs surfaces or AlAs/AlGaAs heterostructures. The Al droplet material is deposited with coverage between one and three monolayers (MLs) at conditions where the As flux is reduced by a factor of about 100 in comparison to conventional layer-by-layer growth of GaAs. For this, the MBE chamber is equipped with a valved-cracker cell for As${}_{4}$ evaporation. The planarly deposited Al forms self-assembled droplets in Volmer–Weber growth mode for a minimization of the surface and interface energies. The As then diffuses from the crystalline substrate into the liquid droplets, driven by the concentration gradient. Consequently, the substrate liquefies at the interface to the droplets. Finally, the droplet material is removed by spreading over the substrate surface, and nanoholes are formed. More details of the LDE process and mechanism have been discussed in previous articles (e.g., [27,33,34,35]). For QD generation, the LDE nanoholes are filled by deposition of a GaAs amount corresponding to film thickness of 0.3 to 0.6 nm of GaAs. The deposited GaAs partially fills the nanoholes, driven by capillarity. Importantly, the QD size is defined by the precisely controlled filling level, which results in highly uniform QD ensembles with well-controlled emission energy [19]. The surface is then capped by AlGaAs.

**Figure 1:**
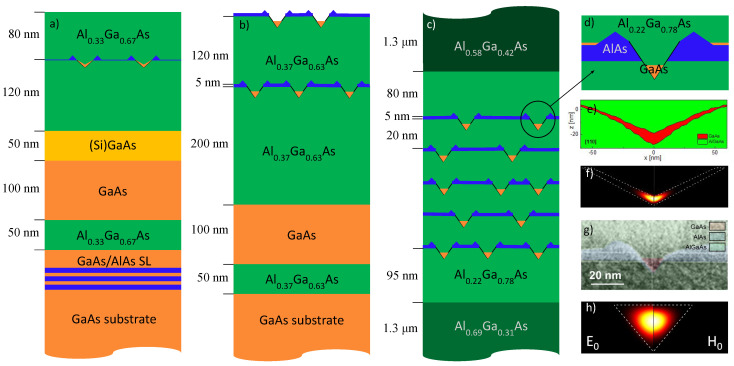
Cross-section schematics of samples (**a**) type I, (**b**) type II and (**c**) type III; (**d**) magnified schematic of a single quantum dot (QD) of type III with surroundings; (**e**) atomic force microscopy (AFM) linescans of the central part of a type I GaAs V-shaped QD in AlGaAs from a sample series illustrating the different interfaces during QD fabrication [37,38]; (**f**) cross-sectional simulated probability densities of the electron $E_{0}$ (left) and hole $H_{0}$ (right) ground states in a V-shaped QD [37,38]; the simulations are performed using a finite-element approach basing on Schrödinger equation in effective-mass approximation; (**g**) cross-sectional transmission electron microscopy (TEM) image of a GaAs cone QD fabricated by filling a nanohole in AlAs/AlGaAs [36]; the color-coding of the materials is a guide for the eyes; (**h**) cross-sectional simulated probability densities in a cone [37,38].

The density of the droplet-etched QDs is determined using atomic force microscopy (AFM, Veeco Dimension 3200, MI, USA) in tapping mode. For an evaluation of the QD shape, we use cross-sectional transmission electron microscopy (TEM, 300 kV JEOL 3010, MA, USA) [36] (Figure 1g) and AFM linescans [37,38] (Figure 1e). The AFM linescans are taken from different samples; shifts of the process parameters can cause fluctuations and the AFM linescans are assumed to show the general shape of the QDs, but the layer thicknesses are possibly not precise.

Three sample designs with GaAs LDE QDs are discussed and will be denoted as type I (single layer of low-density QDs in thin AlGaAs), type II (single layer of high-density QDs in thin AlGaAs) and type III (5 layers of high-density QDs in thick AlGaAs). The thickness and the composition of the respective layers is controlled by a calibration of the GaAs and AlAs growth speed using reflection high-energy electron diffraction (RHEED-12, STAIB Instruments GmbH, Munich, Germany) oscillations.

*Type I:* Standard sample design for single-dot spectroscopy (Figure 1a). After GaAs oxide removal by heating at 600 ${}^{\circ }C$, an AlAs/GaAs superlattice (SL) is grown in order to smoothen the surface. In the next step, an Al${}_{0.33}$Ga${}_{0.67}$As buffer layer of thickness 50 nm is deposited on the GaAs substrate, followed by GaAs (100 nm), (Si)GaAs (50 nm) and Al${}_{0.33}$Ga${}_{0.67}$As (120 nm). The highly doped (Si)GaAs provides a contact layer for field and charge control via a gate electrode. In the present experiments discussed here, no gate electrode was used and the contact layer was floating. The As valve is then closed and one ML of Al is deposited at a AlAs flux of 0.4 ML/s at 600 ${}^{\circ }C$, which yields Al droplets with a density of $2\;\times \;10^{7}$ cm^−2^ [39] in Volmer–Weber mode. A post-growth annealing step of 180 s at $T=620\;{}^{\circ }C$ follows, during which the Al droplets transform into self-assembled nanoholes surrounded by AlAs walls. For the formation of QDs, the holes are then filled with GaAs in a growth-interrupt manner [40] (Ga flux: 0.8 ML/s, 4 pulses of 0.5 s each, with a pause of 10 s in between) and covered by an Al${}_{0.33}$Ga${}_{0.67}$As layer of thickness 80 nm.

*Type II:* QDs in AlAs/AlGaAs for higher density (Figure 1b). Here, an AlAs layer is deposited before the Al droplet formation, resulting in higher dot densities. A layer of Al${}_{0.37}$Ga${}_{0.63}$As (50 nm) is grown on the GaAs substrate, followed by GaAs (100 nm) and Al${}_{0.37}$Ga${}_{0.63}$As (200 nm). On top of this, 5 nm of AlAs is deposited. The As valve is then closed and Al is deposited for 6 s at a flux of 0.47 ML/s at 650 ${}^{\circ }C$, obtaining Al droplets of density $4\;\times \;10^{8}$ cm^−2^. The QDs are then formed in the same way as for type I, but with 5 pulses of Ga (same flux, duration and pause). This technique is known to result in slightly higher QD densities [40]. The surface is covered with a 120 nm thick Al${}_{0.37}$Ga${}_{0.63}$As layer. The steps from the deposition of the AlAs to the filling of the nanoholes with GaAs are then repeated for AFM analysis.

*Type III:* Stack of 5 layers of high-density QDs in AlAs/AlGaAs with thick AlGaAs layer (Figure 1c,e). Following the growth of the GaAs buffer layer, 1.3 μm of Al${}_{0.31}$Ga${}_{0.69}$As, 95 nm of Al${}_{0.23}$Ga${}_{0.77}$As and 5 nm of AlAs are deposited. With the As valve closed, Al droplets are deposited with the same parameters as for type II, thus obtaining nanoholes through the previously described process. These are filled with GaAs (Ga flux: 0.88 ML/s, 5 pulses of 0.5 s with a pause of 10 s between each pulse) and covered by 20 nm Al${}_{0.23}$Ga${}_{0.77}$As. The growth sequence, from the AlAs deposition to the capping of the QDs, is repeated 5 times, leading to 5 layers of high-density ($4\;\times \;10^{8}$ cm^−2^) QDs separated by 20 nm of Al${}_{0.23}$Ga${}_{0.77}$As. Additional 60 nm of Al${}_{0.23}$Ga${}_{0.77}$As, 1.3 μm Al${}_{0.58}$Ga${}_{0.42}$As and 10 nm of GaAs are then deposited.

### 2.2. Photoluminescence Spectroscopy

The optical emission of the LDE QDs is studied using photoluminescence (PL) spectroscopy. Two setups are used, one for single-dot micro-PL at cryogenic temperatures and the other, equipped with a Peltier thermoelectric cooling-stage with a minimum temperature of about 200 K, is used for high-*T* measurements at and close to $T_{R}$.

Both setups are equipped with a focused laser for excitation at 532 nm, the high-*T* setup has also an additional blue laser at 405 nm. The minimum laser spot diameter $D_{L}$ for the high-*T* setup depends on the objective lens with $D_{L}=764$ nm for the 100× lens and $D_{L}=865$ nm for 50×. Thus, for low-density type I QDs, on average, 0.09 (100×) and 0.12 (50×) dots fall within the laser spot; hence, single-dot lines are expected to be measurable. For high-density type II dots, on average, 1.8 (100×) and 2.3 (50×) dots are estimated within the laser spot. The 5× stack (type III) has five times higher dot density than type II within the focus. Thus, for type III sample, the ensemble PL is measured.

In reality, due to the imperfect focusing and lateral diffusion of excited charge carriers, a higher number of excited dots is expected. A further difference between type I and type II/III QDs is related to the shape of the dots. Type I QDs in AlGaAs are V-shaped (Figure 1e) [37] due to the small side-facet angle of about 28° of the initial nanoholes in the AlGaAs surface. Simulations of the electron and hole probability densities in such dots indicate a disk-like shape of the wave-functions (Figure 1f) [37,38]. On the other hand, nanoholes in AlAs have much steeper side-facets (Figure 1g), with angles of about 50° [37]. Accordingly, the shape of type II/III QDs is cone-like and the simulated wave functions are close to a sphere (Figure 1h) [37,38]. As a consequence of the difference in shape, the confinement potential is expected to be stronger in type II and type III samples as opposed to type I. In type III, besides the geometry, the lower Al content further influences the potential, making the effective exciton dissociation energy the lowest between the three sample types.

## 3. Results and Discussion

### 3.1. Influence of Sample Temperature

This section discusses PL data from the three sample types as function of sample temperature. The data are taken using a green laser (532 nm).

**Figure 2:**
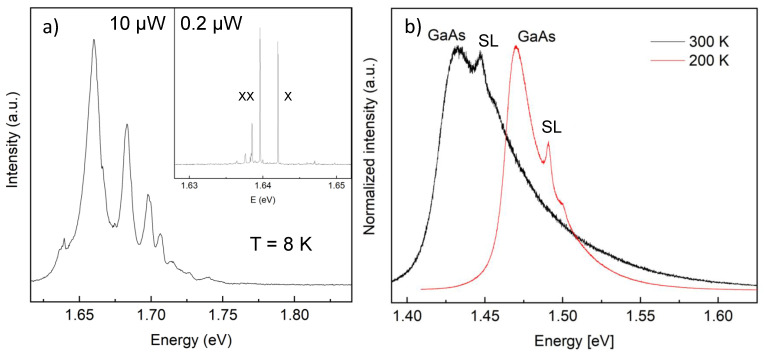
Photoluminescence (PL) data from sample type I taken with a green laser (532 nm). (**a**) Single-dot PL spectrum at low T=8 K with 10 μW laser power; inset: PL emission from a single QD with 0.2 μW excitation power, exciton (X) and biexciton (XX) peaks are clearly visible; (**b**) PL spectrum at $T_{R}$ (black line) and at 200 K (red line) with 0.06 mW excitation power. The labels denote the emission from the GaAs substrate (GaAs) and from the superlattice (SL).

*Type I*: As reference, single-dot PL measurements of a low-density type I sample are first analyzed at cryogenic temperature (T=8 K). At low-excitation power of 0.2 μW, the exciton (X) and biexciton (XX) peaks are sharp and clearly visible (inset of Figure 2a), with linewidth of about 50 μeV. At higher excitation power of 10 μW, the higher QD energy levels are also occupied, and the single-dot PL lines clearly show well-separated ground state and three excited states. The lines are broadened due to the formation of multiexcitonic complexes (Figure 2a). Emission from the ground-state exciton is observed at $E_{0}$ = 1.642 eV. We note that no background from the GaAs substrate is visible at the cryogenic temperatures. Using the well-known Varshni relation, we estimate for the GaAs bandgap energy at *T* = 8 K a value of $E_{g}$ = 1.519 eV. This indicates a quantization energy for the QD ground-state of $E_{0}-E_{g}$ = 123 meV. For the high-*T* measurements, we extrapolate an energy of $E_{0}\left(T=200\;K\right)$ = 1.588 eV and $E_{0}\left(T=300\;K\right)$ = 1.545 eV.

For the high-temperature measurements, a sample stage with position control is used and an $8\times 8\;\mu m^{2}$ sample area is scanned with 500 nm step size. From the QD density of $2\;\times \;10^{7}$ cm^−2^, as determined by AFM, 13 QDs are expected on average within the scanned sample area. The PL emission (Figure 2b) is similar for all scanned sample fields and shows a very broad asymmetric peak and a second peak at a higher energy (16 meV at 300 K and 21 meV at 200 K). The broad asymmetric peak is caused by the GaAs substrate and will be discussed below in more detail. We attribute the peak at higher energy to quantized states in the AlAs/GaAs-superlattice. Most importantly here, no indication of QD emission at or close to the expected energy $E_{0}$ is observed for both temperatures and for all scanned sample fields. Even subtraction of the GaAs background does not resolve clear QD signals.

**Figure 3:**
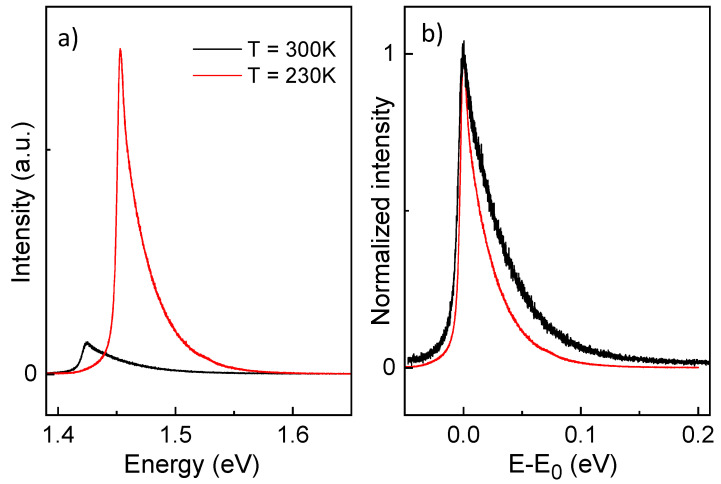
(**a**) PL data from a GaAs wafer taken with a green laser (532 nm) at T=300 K (black) and T=230 K (red). (**b**) Normalized GaAs PL intensity as a function of the difference between emitted energy and GaAs band-gap energy.

The strong and undesired background at high temperatures can be related to emission from the GaAs substrate. From the PL emission of a GaAs wafer at T=300 K and 230 K (Figure 3a), various features can be inferred. The PL signal is very broad and shows a strong high-energy tail, which broadens with increasing *T*. The tail and its *T*-dependence reflect the thermal population of high-energy states (band-to-band recombination of free carriers) [41] and massively interferes with possible emissions at the expected QD energies, unlike in the low-*T* PL. The maximum of the PL spectrum approximately agrees with the GaAs band-gap energy. It can be also noted that the PL intensity is decreasing with increasing *T*; this can be explained by a combined effect of exciton dissociation, which reduces the coupling to the light mode, and thermally activated non-radiative decay mechanisms.

*Type II*: For the next sample type, a number of improvements have been considered for the enhancement of the QDs’ optical signal. As a central point, the QD density is increased by a factor of 20, from $2\;\times \;10^{7}$ cm^−2^ to $4\;\times \;10^{8}$ cm^−2^. This is achieved by an additional AlAs layer (Figure 1b), which modifies the surface diffusion of the deposited Al adatoms, causing a higher density of droplets, and thus of QDs [26]. Furthermore, the thickness of the AlGaAs barrier layer is increased from 200 nm to 320 nm, its Al content from 33% to 37%, and the (Si)GaAs back gate is removed.

In previous ensemble PL measurements [19,26], low-temperature QD ground-state emission is observed at 1.578 eV (T=4 K). From the temperature-dependent shift of the GaAs band-gap, the QD PL emission is expected at 1.525 eV for T=200 K and 1.481 eV for T=300 K. For the PL acquisition, the sample surface is scanned as described above for sample type I.

**Figure 4:**
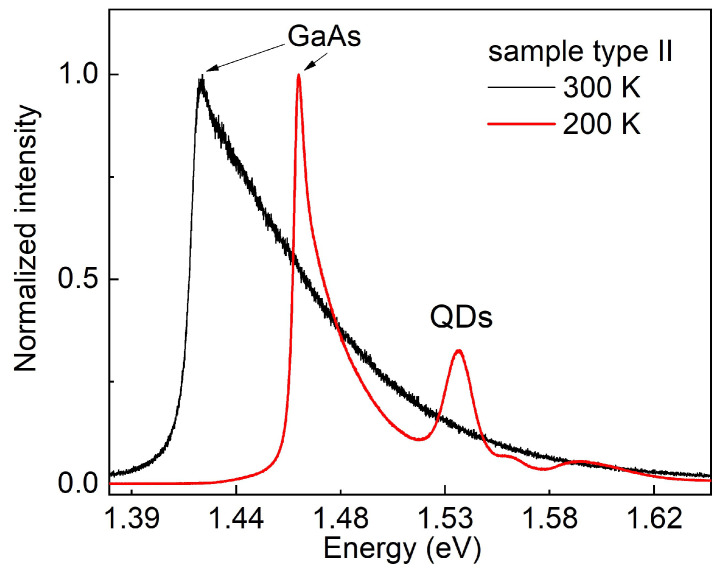
PL data from a type II sample taken with a green laser (532 nm) at $T_{R}$ (black) and 200 K (red) with 0.06 mW excitation power. The ground state emission $E_{0}=1.534\;\mathrm{eV}$ from QD ensemble is visible at 200 K.

In the PL measurement from a type II sample at T=300 K, the strong GaAs background is still dominant and no indication of QD emission is visible (Figure 4, black line), which is typical for most scan fields. For a few scan fields, a very weak shoulder close to the expected QD emission energy may possibly be related to QDs; in these scan fields, the QD density is assumed to be locally higher and the emission visible is associated to a QD ensemble. Hence, at T=300 K, the type II samples are just below the threshold of QD visibility. In contrast, at T=200 K, a clear peak is visible at $E_{0}=1.534\;\mathrm{eV}$ on all scan fields (Figure 4, red line). Since the peak energy is close to the expected QD emission of 1.525 eV, we attribute the peak to the QD ground-state. The slightly higher measured energy can be associated with the QD size uniformity over the wafer. The weak shoulders at 1.558 eV and 1.590 eV can be related to QD excited states. The ground-state quantization energy becomes $E_{0}-E_{g}=68\;\mathrm{meV}$.

*Type III:* To avoid the GaAs background signal, a type III sample has a very thick (over 2.8 μm) AlGaAs barrier (Figure 1c). Moreover, to strengthen the signal coming from the QDs, five layers of QDs analogous to those of sample II are stacked on top of each other.

**Figure 5:**
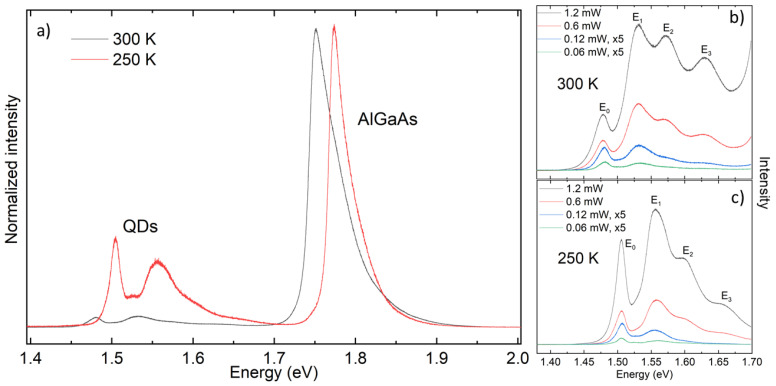
PL data from a type III sample taken with a green laser (532 nm). (**a**) PL spectrum at $T_{R}$ (black) and 250 K (red) with 0.06 mW excitation power; strong QD peaks are visible. (**b**) Zoom of QD spectra at $T_{R}$ and (**c**) at 250 K, taken at different laser powers as indicated. The ground state and up to 3 excited states are clearly visible. The intensity is normalized for equal peak intensities of the luminescence from the AlGaAs barrier.

PL spectra at $T_{R}$ (Figure 5a, black line) show a substantial improvement with respect to the previous samples. No GaAs substrate peak is visible and a very clear emission from the dots can be detected. The desired GaAs peak suppression is caused by the thick AlGaAs region on top of the GaAs substrate. Since the AlGaAs layer is optically active, a strong peak at E=1.75eV is visible. The Al concentration of 22.3% is determined from the AlGaAs peak position. The AlGaAs peak is asymmetric with a high-energy tail, similar to the GaAs bulk peak (Figure 3). However, being well separated from the QD emission, it does not obscure the signal coming from the QDs from 1.4 eV to 1.7 eV.

In the $T_{R}$ PL signals of the QDs (Figure 5b), at high excitation power of 1.2 mW, emission from the ground state and three excited states are visible at 1.479 eV, 1.531 eV, 1.574 eV and 1.632 eV, respectively. After reducing the excitation power to 0.6 mW, four peaks are still visible, although less pronounced. Further reducing the excitation power, the PL emission intensity gradually reduces, in particular for high energy levels. This can be explained by the fact that fewer charge carriers reach the higher excited states.

Analogous measurements have been performed with the sample cooled to 250 K (Figure 5a, red line, and c). The QD peaks become sharper and are blue-shifted by about 27 meV, in agreement with the temperature-dependent shift of the GaAs band-gap energy. Their dependence on the excitation power is similar to the behavior at $T_{R}$. The PL data indicate also a strongly reduced intensity at $T_{R}$ in comparison with the cooled samples (see below).

For an evaluation of the mechanism behind the reduction in the PL intensity with increasing temperature, the quantized energy levels inside the QDs are estimated. The roughly constant spacing ΔE≃51±8 meV between the QD emission energies suggests a 3D simple harmonic oscillator approximation for the confining potential. Under this assumption and considering an idealized spherical dot shape, the electron energy of the $n^{th}$ level ($n=n_{x}+n_{y}+n_{z}=0,1,2,\ldots$, with $n_{i}=0,1,2,\ldots$ quantum numbers) is

(1) $$E_{e,n}=E_{e,n_{x}}+E_{e,n_{y}}+E_{e,n_{z}}=\hbar \omega_{e}\left(n+\frac{3}{2}\right).$$

with $\omega_{e}$ the frequencies of the harmonic oscillator potential. With an analogous argument, the hole energy is

(2) $$E_{h,n}=\hbar \omega_{h}\left(n^{\prime }+\frac{3}{2}\right)$$

with quantum numbers $n^{\prime }=n_{x}^{\prime }+n_{y}^{\prime }+n_{z}^{\prime }=0,1,2\ldots$.

In an ideal case, $\omega_{e}$ and the oscillator length *L* are related as $\omega_{e}=\frac{4\hbar }{m_{e}^{*}L^{2}}$, with $m_{e}^{*}$ the electron effective mass. Using this relation, for allowed optical transitions between electron and hole states with equal quantum numbers $n_{i}=n_{i}^{\prime }$, the electron and hole quantization energy ratio reduces to

(3) $$\frac{E_{e,n}}{E_{h,n}}=\frac{m_{h}^{*}}{m_{e}^{*}}.$$

The PL energy of the $n^{th}$ level is

(4) $$E_{n}=E_{g}+E_{e,n}+E_{h,n}-E_{B}$$

with GaAs band-gap energy $E_{g}$ and electron-hole binding energy $E_{B}$, which is assumed to be independent of $n_{i}$. Combining Equations (3) with (4), the electron and hole energies are given by

(5) $$E_{e,n}=\frac{E_{n}-E_{g}+E_{B}}{\left(1+m_{e}^{*}/m_{h}^{*}\right)}≃0.88\left(E_{n}-E_{g}+E_{B}\right)$$

(with $m_{e}^{*}$ = 0.066 $m_{e}$ and $m_{h}^{*}$ = 0.5 $m_{e}$ for GaAs) and

(6) $$E_{h,n}≃0.12\left(E_{n}-E_{g}+E_{B}\right).$$

Using Equations (1), (2) and (4), the two following energy differences are obtained: $E_{1}-E_{0}=\hbar \left(\omega_{e}+\omega_{h}\right)$ between the first excited state and the ground state and $E_{0}-E_{g}=\frac{3}{2}\hbar \left(\omega_{e}+\omega_{h}\right)-E_{B}$ between the ground state and the band-gap energy. Combining and rearranging these two expressions, the binding energy is $E_{B}=E_{g}-\frac{5}{2}E_{0}+\frac{3}{2}E_{1}$. The numerical value of the binding energy can thus be obtained through the experimental results as $E_{B}=23\;\mathrm{meV}$. The oscillator length corresponding to $E_{B}=23\;\mathrm{meV}$ is L≃10 nm can give an indication of the order of magnitude of the QD diameter and it is in accordance with morphological observations of type III QDs, such as the TEM image shown in Figure 1g.

**Figure 6:**
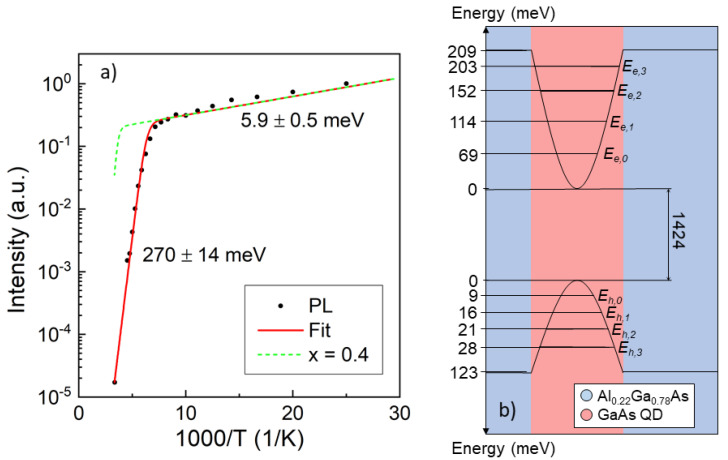
PL data from a type III sample taken with a green laser (532 nm). (**a**) Comparison of measured PL ground-state intensities at different temperatures (symbols) with model results (red line) using the indicated activation energies. The dashed green line is an estimation using the same model but for a higher Al content of 40%. (**b**) Diagram of conduction and valence band at the interface between Al${}_{0.22}$Ga${}_{0.78}$As barrier (blue) and GaAs QD (red). The QD energy levels and the band-gap energies at $T_{R}$ are indicated; all the energy values are in meV.

Using Equations (5) and (6), the $n^{th}$-level electron and hole energies can now be determined from the PL data (Table 1). Measurements of the integrated PL intensity of the QD ground state with 2 μW excitation power and at varied temperature show a substantial intensity decrease by a factor of 1/200, with *T* increasing from T=40 K up to T=300 K (Figure 6a). A detailed analysis indicates three regimes, from 40 K to 100 K (low-temperature regime), from 100 K to 160 K (transition regime) and for temperatures above 160 K (high-temperature regime) (Figure 6a). Assuming thermally activated processes to be responsible for the reduction in the emission energy, the activation energies can be determined from the slopes of the plot in Figure 6a. For the transition regime, a single slope cannot be assigned, whilst for the low- and high-temperature regimes, a clear slope is visible. The energies that best fit the temperature dependence are $E_{A1}=5.9\;\mathrm{meV}$ and $E_{A2}=270\;\mathrm{meV}$ for the low- and high-temperature regimes, respectively.

It can be noted that the low-temperature activation energy $E_{A1}=5.9\;\mathrm{meV}$ is close to the exciton binding energy of 4.7 meV in GaAs bulk material [42]. This would suggest that, at temperatures between 40 K and 100 K, excitons excited by the laser in the AlGaAs barrier break in a thermally activated process, and the resulting single charge carriers separately diffuse through the crystal.

The high-temperature activation energy $E_{A2}=270\;\mathrm{meV}$ can be associated with various loss channels within the QD. In the following, the most likely mechanisms are proposed, along with the energies needed for each transition in order to compare the values with $E_{A2}$.

*Exciton escape*: an electron and a hole escape together as an exciton into the AlGaAs barrier material without breaking the Coulomb binding-energy $E_{B}$; in this case, the escape energy is $E_{X,esc}=\Delta E_{c}-E_{e,n}+\Delta E_{v}-E_{h,n}$, where $\Delta E_{c}$ and $\Delta E_{v}$ are the band discontinuities at the AlGaAs/GaAs interface for the conduction band and the valence band, respectively. The values used for the band discontinuities for AlGaAs with an Al content of x=0.223 are $\Delta E_{c}=0.209\mathrm{eV}$ and $\Delta E_{v}=0.123\mathrm{eV}$ [43].

*Single charge escape*: the excitonic bond between electrons and holes is broken and one charge carrier escapes separately into the conduction or valence band. The escape energy becomes $E_{e,esc}=\Delta E_{c}-E_{e,n}+E_{B}$ for the electrons and $E_{h,esc}=\Delta E_{v}-E_{h,n}+E_{B}$ for the holes.

The results for the different processes are summarized in Table 1. To identify the corresponding escape mechanism, $E_{A2}=270\;\mathrm{meV}$ is compared with the different escape energies given in Table 1. Within the limits of the experimental accuracy, $E_{A2}$ can be associated with the escape of ground-state bound exciton into the barrier material ($E_{X,esc}=254\;\mathrm{meV}$).

The model shown in Figure 6a, closely fitting the experimental data, is based on the following rate model. The optical intensity emitted from a QD is $I_{PL}=N_{QD}R_{PL}$, with $N_{QD}$ the exciton population in the QD and $R_{PL}=\frac{1}{\tau_{PL}}$ the rate at which an exciton radiatively recombines in the QD, with radiative lifetime $\tau_{PL}$. The change in time of the exciton population in the QD is $\frac{dN_{QD}}{dt}=N_{B}R_{cap}-N_{QD}R_{esc}-N_{QD}R_{PL}$, with $N_{B}$ the exciton population in the barrier material, $R_{cap}$ the rate at which an exciton from the barrier is captured by the QD, and $R_{esc}$ the rate at which an exciton thermally escapes from the QD. At constant illumination, $\frac{dN_{QD}}{dt}=0$ and $N_{QD}=\frac{N_{B}R_{cap}}{R_{esc}+R_{PL}}$. The change in exciton population in the barrier material is $\frac{dN_{B}}{dt}=R_{E}-N_{B}R_{br}$; at constant illumination, $\frac{dN_{B}}{dt}=0$ and thus $N_{B}=\frac{R_{E}}{R_{br}}$. This gives $N_{QD}=\frac{R_{E}R_{cap}}{R_{br}\left(R_{esc}+R_{PL}\right)}$, which yields:

(7) $$I_{PL}=\frac{R_{E}R_{cap}R_{PL}}{R_{br}\left(R_{esc}+R_{PL}\right)}.$$

It is now assumed that $R_{br}=\nu_{br}\exp\left(-E_{A1}/k_{B}T\right)$ and $R_{esc}=\nu_{esc}\exp\left(-E_{A2}/k_{B}T\right)$ are thermally activated rates with vibraional frequencies $\nu_{br}$ and $\nu_{esc}$ and activation energies $E_{A1}$ and $E_{A2}$, whereas $R_{E}$, $R_{cap}$ and $R_{PL}$ are *T*-independent. Then, Equation (7) simplifies to

(8) $$I_{PL}\propto \frac{1}{R_{br}\left(R_{esc}+R_{PL}\right)}.$$

A comparison of model results calculated using $\tau_{PL}=1\;\mathrm{ns}$, $E_{A1}=5.9\;\mathrm{meV}$ and $E_{A2}=270\;\mathrm{meV}$ with measured PL ground state intensities is shown in Figure 6. The very good agreement supports the validity of the model.

### 3.2. Influence of Laser Energy

This section discusses PL data from the three sample types measured using a blue laser (405 nm) for excitation. The improved contrast between QD emission and GaAs background can be estimated using a simple picture considering the wavelength dependent absorption coefficient α of the AlGaAs barrier material: α(532 nm) = $5.59\;\times \;10^{4}$ cm^−1^ and α(405 nm) = $4.19\;\times \;10^{5}$ cm^−1^ for an Al content of 31.5% [44]. Thus, the absorption coefficients at 405 nm and 532 nm differ by almost an order of magnitude. Accordingly, the ratio of the excitation intensities at the depth $d_{QD}$ = 80 nm of the dot plane is I(405 nm)/I(532 nm) = $5\;\times \;10^{-2}$ and at the depth $d_{GaAs}$ = 200 nm of the interface between AlGaAs barrier and GaAs substrate I(405 nm)/I(532 nm) = $7\;\times \;10^{-4}$. One thus may expect a suppression of the substrate luminescence at 405 nm excitation wavelength by a factor of roughly 70, if other effects like diffusion of excitons do not render this estimation invalid. In summary, a blue laser is expected to cost QD excitation intensity but to increase the contrast $I_{QD}/I_{GaAs}$ between QD and GaAs emission by a factor of 70.

**Figure 7:**
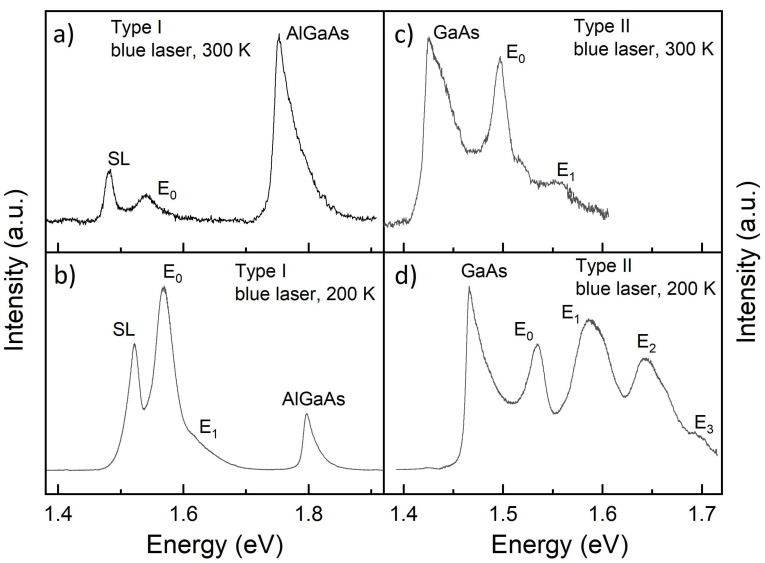
PL data from type I and type II samples taken with a blue laser (405 nm). (**a**) Type I, *T* = 300 K, (**b**) type I, *T* = 200 K, (**c**) type II, *T* = 300 K, (**d**) type II, *T* = 200 K. The labels, GaAs and AlGaAs, denote the substrate and barrier bulk-peaks, SL the AlAs/GaAs superlattice, and $E_{0}$, $E_{1}$, … the QD states.

*Type I*: Figure 7a,b show PL data from a type I sample for excitation with a blue laser. The sample temperatures are *T* = 300 K and *T* = 200 K, respectively. Both spectra look fundamentally different in comparison to the data obtained with the green-laser excitation (Figure 2). Here, no peak at the GaAs band-gap energy is visible, which supports the above estimation of a suppressed excitation of the GaAs substrate. The SL peaks are visible at both temperatures as well as the peak from the AlGaAs barrier; both are also visible with the green laser (not shown in Figure 2). As a major improvement of the blue laser, now a weak QD peak (E${}_{0}$) is visible already at *T* = 300 K. The energy of $E_{0}$ = 1.540 eV indicates a quantization energy $E_{0}-E_{g}$ = 118 meV, which is close to the value of 123 meV estimated above for the measurements with a green laser at *T* = 8 K. For *T* = 200 K, the intensity of the QD and of the SL increase above that from the AlGaAs barrier and a weak shoulder at about 1.626 eV can indicate an excited state.

*Type II*: PL data from a type II sample with excitation by a blue laser are shown in Figure 7c,d. At *T* = 300 K, a weak GaAs peak is visible and already a QD signal with a clear ground-state peak E${}_{0}$ and a very weak excited state E${}_{1}$. This is again superior in comparison to excitation with a green laser. The ground-state quantization energy is $E_{0}-E_{g}$ = 71 meV, which agrees well with the value of 68 meV obtained above using a green laser at *T* = 200 K. As a further noticeable point, the intensity of the GaAs peak is reduced down to 0.016 % in comparison to measurements with a green laser for excitation. At *T* = 200 K and the same excitation intensity, the QD signal becomes much stronger with one ground-state and three excited-state peaks. The ground-state quantization energy is $E_{0}-E_{g}$ = 68 meV, which is close to the room-temperature result.

*Type III*: PL measurements of a type III sample with a blue laser at *T* = 300 K and *T* = 200 K show no QD-related emission (the data are not shown). This is in contrast to the spectra obtained using a green laser and can be explained by the very thick and, thus, highly absorbing AlGaAs barrier in this sample.

## 4. Conclusions

The results presented in this work demonstrate that for the investigation of room-temperature emission from quantum dots, a layer design is recommended with which the otherwise strong contribution of the substrate luminescence can be avoided. Samples with a thin AlGaAs barrier layer on top of the substrate show a very strong and broad GaAs substrate emission which covers the QD signals. Here, an optimized sample design with an AlGaAs layer thicker than the penetration depth of the exciting laser light yields a substantial improvement and demonstrates clear QD peaks already at room temperature. The optical penetration depth can be also reduced by using a blue laser at 405 nm for excitation instead of a usual green laser at 532 nm. With a blue laser, weak QD peaks at room temperatures are visible also for samples with a thin AlGaAs barrier. However, the excitation intensity and, thus, the QD emission is strongly reduced due to the higher absorption.

As a further important point, the temperature-dependent analysis of the integrated QD PL intensity indicates a decrease in intensity by more than four orders of magnitude for room temperature in comparison to experiments carried out at liquid helium temperature. Through a rate model, assuming thermally activated loss channels, the activation energies are fitted for a low- and a high-temperature regime. The analysis suggests that, at temperatures below 100 K, the intensity loss can be associated with thermally activated dissociation of laser-excited excitons in the AlGaAs barrier and the corresponding formation of single-charge carriers diffusing separately through the crystal. These broken excitons would not contribute to the QD PL emission. At temperatures above 160 K, the loss could be caused by bound excitons escaping from the ground state into the barrier material. We note that also non-radiative recombination by Auger-type processes increases with temperature [45]. However, ref. [45] shows that these processes become strong only at very high excitation densities. We assume that our excitation conditions are below that regime.

The exciton dissociation process at low *T* would be avoided by using a different pumping mechanism, such as electrical injection and resonant or quasi-resonant pumping into the QD. Furthermore, since an electric field is expected to increase the charge carrier separation, it would be advisable to place the QDs further away from the surface to avoid possible charge-induced fields. To reduce the loss of PL intensity in the high temperature regime, it is recommended to increase the barrier height by choosing an AlGaAs barrier with a higher Al content. The maximum reasonable Al content in the AlGaAs barrier is limited to 40% to avoid an indirect band structure. The improvement by a higher barrier is demonstrated in Figure 6a, where a calculation using the above model for sample type III but for a higher Al content of 40% predicts an increase in the QD intensity at room temperature by more than three orders of magnitude.

Moderate thermoelectric cooling using an inexpensive Peltier element strongly enhances the QD emission. Here, the two major issues are already considerably improved. First, the linewidth of the GaAs substrate peak is smaller, which reduces the substrate background at the QD emission energy. Second, the intensity loss by thermal escape of excitons from the dots is reduced yielding a stronger QD emission. This is demonstrated for instance in Figure 7a,b, where thermoelectric cooling down to 200 K increases the QD ground-state intensity by a factor of about 200.

## Figures and Tables

**Table 1:** Measured PL energies $E_{n}$ for QD sample type III at $T_{R}=300\;K$. Calculated electron and hole quantization energies and escape energies. All energy values are in meV.

Level, *n*	0	1	2	3
$E_{n}$	1479	1531	1574	1632
$E_{n}-E_{n-1}$	-	52	43	58
$E_{e,n}$	69	114	152	203
$E_{h,n}$	9	16	21	28
Exciton escape				
$E_{X,esc}$	254	202	159	101
Single charge escape				
$E_{e,esc}$	163	118	80	29
$E_{h,esc}$	137	130	125	118

## Data Availability

Not applicable.

## Abbreviations

The following abbreviations are used in this manuscript:

QDs	Quantum dots
$T_{R}$	Room temperature
PL	Photoluminescence
MBE	Molecular beam epitaxy
LDE	Local droplet etching
ML	Monolayers
AFM	Atomic force microscope
TEM	Transmission electron microscopy
SL	Superlattice
